# Integrated Clinical and Genomic Models to Predict Optimal Cytoreduction in High-Grade Serous Ovarian Cancer

**DOI:** 10.3390/cancers14143554

**Published:** 2022-07-21

**Authors:** Nicholas Cardillo, Eric J. Devor, Silvana Pedra Nobre, Andreea Newtson, Kimberly Leslie, David P. Bender, Brian J. Smith, Michael J. Goodheart, Jesus Gonzalez-Bosquet

**Affiliations:** 1 Department of Obstetrics and Gynecology, University of Iowa, 200 Hawkins Dr., Iowa City, IA 52242, USA; eric-devor@uiowa.edu (E.J.D.); silvana-pedranobre@uiowa.edu (S.P.N.); david-bender@uiowa.edu (D.P.B.); michael-goodheart@uiowa.edu (M.J.G.); jesus-gonzalezbosquet@uiowa.edu (J.G.-B.); 2 Nebraska Medical Center, Division of Gynecologic Oncology, University of Nebraska, Omaha, NE 68198, USA; anewtson@unmc.edu; 3 Departments of Internal Medicine and Obstetrics and Gynecology, Division of Molecular Medicine, The University of New Mexico Comprehensive Cancer Center, 915 Camino de Salud, CRF 117, Albuquerque, NM 87131, USA; kkleslie@salud.unm.edu; 4 Department of Biostatistics, University of Iowa, 145 N Riverside Dr., Iowa City, IA 52242, USA; brian-j-smith@uiowa.edu

**Keywords:** high-grade serous carcinoma, ovarian cancer, surgical cytoreduction, prediction modeling

## Abstract

**Simple Summary:**

Approximately 30% of patients with advanced, high-grade serous ovarian cancer who undergo surgery will have a suboptimal result, resulting in decreased overall survival. Improving the ability to predict a successful surgery would improve survival. We aimed to use tumor genomics to create prediction models, which would predict an optimal or complete cytoreduction prior to entering the operating room. We created two sets of models, one for optimal and one for complete cytoreduction. We then validated those models using the TCGA database as well as statistical learning. We developed 21 models for optimal cytoreduction and 37 models for complete cytoreduction, which have the potential to improve our ability to predict these surgical results in patients with ovarian cancer before taking them to the operating room. Improving our pre-operative decision-making will result in more patients having the desired surgical results and, therefore, improved survival.

**Abstract:**

Advanced high-grade serous (HGSC) ovarian cancer is treated with either primary surgery followed by chemotherapy or neoadjuvant chemotherapy followed by interval surgery. The decision to proceed with surgery primarily or after chemotherapy is based on a surgeon’s clinical assessment and prediction of an optimal outcome. Optimal and complete cytoreductive surgery are correlated with improved overall survival. This clinical assessment results in an optimal surgery approximately 70% of the time. We hypothesize that this prediction can be improved by using biological tumor data to predict optimal cytoreduction. With access to a large biobank of ovarian cancer tumors, we obtained genomic data on 83 patients encompassing gene expression, exon expression, long non-coding RNA, micro RNA, single nucleotide variants, copy number variation, DNA methylation, and fusion transcripts. We then used statistical learning methods (lasso regression) to integrate these data with pre-operative clinical information to create predictive models to discriminate which patient would have an optimal or complete cytoreductive outcome. These models were then validated within The Cancer Genome Atlas (TCGA) HGSC database and using machine learning methods (TensorFlow). Of the 124 models created and validated for optimal cytoreduction, 21 performed at least equal to, if not better than, our historical clinical rate of optimal debulking in advanced-stage HGSC as a control. Of the 89 models created to predict complete cytoreduction, 37 have the potential to outperform clinical decision-making. Prospective validation of these models could result in improving our ability to objectively predict which patients will undergo optimal cytoreduction and, therefore, improve our ovarian cancer outcomes.

## 1. Introduction

Ovarian cancer is the leading cause of gynecologic cancer death and the fifth most common cancer in women in the United States. The disease carries a poor prognosis with 5-year overall survival approaching 50%. This is mainly because most cases are diagnosed at an advanced stage, either FIGO Stages III or IV [1].

Ovarian cancer is a heterogeneous disease encompassing several histological subtypes. Epithelial ovarian cancer accounts for approximately 90% of cases and the most common epithelial subtype is high-grade serous carcinoma (HGSC), which is also the deadliest. Treatment of HGSC consists of a combination of surgery and chemotherapy, either with cytoreductive surgery primarily or after 3 to 6 rounds of platinum-based chemotherapy [2].

The Gynecologic Oncology Group (GOG) defines optimal surgical resection (R1) as ‘no residual lesions greater than 1 cm in maximal diameter’, and has used this metric in ovarian cancer clinical trials since 1996, and as recently as 2019 [3,4,5,6,7,8]. If there is no visible residual disease then surgical resection is termed complete (R0) [8]. Overall survival is strongly associated with the amount of residual disease after surgery, with the best survival occurring after complete resection [9,10,11,12,13].

Upfront surgical resection is preferred if an optimal surgical result can be achieved [14]. However, if an optimal or complete resection of the disease is not feasible, neoadjuvant chemotherapy is used to improve the chances of optimal resection without compromising overall survival [15,16,17]. Surgeons traditionally use clinical data, such as physical examination, imaging studies, and functional status to determine the ability to successfully resect disease. In high-volume centers, this leads to an optimal surgical result in 70% of cases [18]. Numerous attempts have been made to try and improve the surgeon’s ability to predict an optimal surgical outcome. Clinical data, such as CA-125, and imaging studies combined into predictive models, have not yielded a formula that improves an experienced surgeon’s decision-making [19,20,21]. A laparoscopic scoring system yielded to a complete resection 88% of the time, but still requires a surgical procedure and increased use of resources [22].

Some believe that tumor biology plays a role in the likelihood of optimal surgical resection and that molecular characterization can improve rates of optimal resection [23]. A previously published gene expression signature accurately categorized suboptimal and optimal outcomes 92.8% of the time [24]. However, a subsequent study concluded that prediction using gene expression was limited by the fact that the surgical outcome was sometimes dictated by intraoperative decision-making based on patient health factors [25]. We hypothesize that integrating genomic data from surgical specimens with clinical data will improve the prediction of optimal cytoreductive surgery in ovarian cancer over clinical decision-making.

We leveraged the University of Iowa (UI) Biobank, which harbors hundreds of high-grade serous cancers (HGSC) of tubo-ovarian or peritoneal origin. We extracted DNA and RNA from these tumors and determined their genomic features: DNA methylation, gene expression, exon expression, micro RNA (miRNA), long non-coding RNA (lncRNA), single nucleotide variation (SNV), copy number variation (CNV), and fusion transcripts. Our objective was to create accurate prediction models that would discriminate which patients with HGSC would undergo optimal or complete cytoreductive surgery using both pre-operative clinical and genomic data. Then, we validated these models using The Cancer Genome Atlas (TCGA), an independent comprehensive clinical genomic database, and using machine learning.

## 2. Materials and Methods

We performed a retrospective, single-institution cohort study in which we included all Stage III and IV patients with high-grade serous ovarian cancer (HGSC) from 1990 to 2014 available in our biobank with pre-operative and intra-operative clinical data. DNA and RNA were then extracted from tumor specimens and processed as detailed below to obtain the necessary genomic data. Clinical and genomic data were then combined to create predictive models using statistical learning to identify criteria that accurately predicted optimal and complete cytoreduction in advanced-stage HGSC patients.

Tissue samples and clinical outcome data were obtained from the Department of Obstetrics and Gynecology Gynecologic Oncology Biobank (IRB, ID no. 200209010), which is part of the Women’s Health Tissue Repository (WHTR, IRB, ID no. 201804817). All of the tissues archived in the Gynecologic Oncology Biobank (herein termed Biobank) were originally obtained from adult patients under informed consent in accordance with University of Iowa IRB guidelines. Tumor samples were collected, reviewed by a board-certified pathologist, flash-frozen, and then the diagnosis was confirmed in paraffin at the time of initial surgery. All experimental protocols were approved by the University of Iowa Biomedical IRB-01.

The University of Iowa is a high-volume clinical center with gynecologic oncologists trained in extensive cytoreductive surgery, including upper abdominal debulking. All procedures were performed by board-certified or board-eligible gynecologic oncologists with the assistance of appropriate consulting services if necessary.

We analyzed patients based on the amount of residual disease at the conclusion of surgery. Traditionally, the objective of surgery has been to achieve optimal cytoreduction, meaning that there are no residual lesions greater than 1 cm in size. If this is not achieved, it is termed suboptimal debulking. If all visible disease is removed at the time of surgery, then that is termed a complete cytoreduction. More recently, as some surgeons have adopted complete cytoreduction as their surgical objective, we chose to create two sets of predictive models: one that predicted optimal cytoreductions, including all patients with residual disease less than 1 cm, which by definition included complete cytoreductions, and the second set of models, which predicted complete cytoreduction only. When referring to the comparison groups for these two sets of models, we will refer to the groups as optimal and suboptimal, and complete and incomplete, respectively.

### 2.1. Clinical Data

Clinical data were extracted from the electronic medical record. Table 1 summarizes the baseline clinical and pathologic characteristics. Only data that were available prior to surgical intervention were used in the development of predictive models, including age, BMI, Charlson Comorbidity index, CA-125, stage, use of neoadjuvant chemotherapy, and location of disease on imaging. Other clinical data were collected to compare outcomes and demonstrate the extent of surgical resection.

Clinical data from the University of Iowa Biobank were also used to determine the rate of optimal cytoreduction through time at our institution to act as our threshold for a successful predictive model.

### 2.2. Genomic Analysis

**Figure 1:**
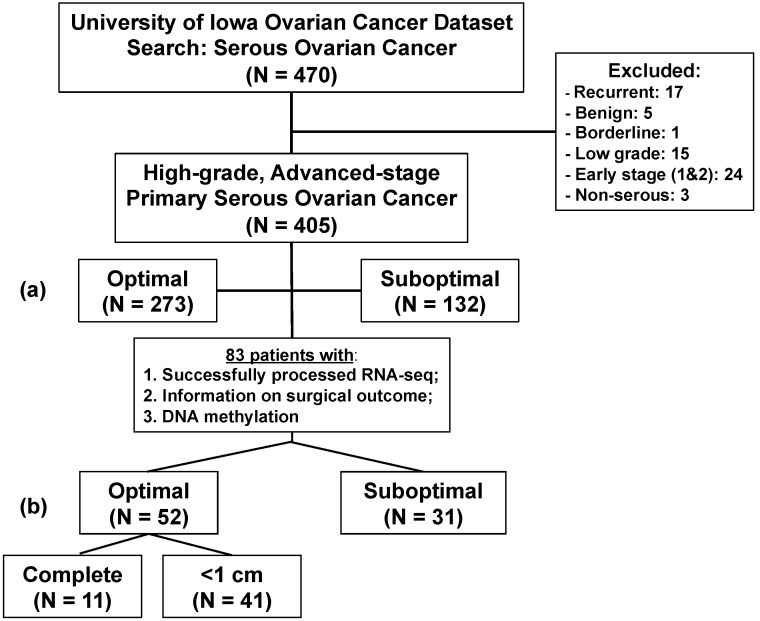
The flow of included patients in the analysis. Of the initial 470 HGSC patients, 405 were confirmed to be high-grade, advanced stage, of serous histology: (**a**) 273 underwent optimal PCS, and 132 underwent suboptimal PCS. A total of 187 had frozen tumors in the biobank, and RNA-seq and DNA methylation was successful in 83: (**b**) 31 underwent suboptimal PCS and 51 underwent optimal PCS, 11 of them (or 22%) with R0 or no macroscopic disease.

A total of 470 patients with ovarian cancer were identified in the database. Only patients with primary diagnoses of advanced stages III and IV, high-grade serous ovarian cancer, were included. The flow diagram in Figure 1 summarizes the patients included in this study.

RNA was then isolated from these tumor specimens. RNA extraction, processing, and sequencing have been described previously [28,29]. In brief, total cellular RNA was extracted from primary tumor tissue using the mirVana (Thermo Fisher, Waltham, MA, USA) RNA purification kit. The RNA yield and quality were assessed with Trinean Dropsense 16 spectrophotometer and Agilent Model 2100 bioanalyzer. RNA quality was determined to be adequate if the sample had an RNA integrity number (RIN) of 7.0 or greater. Samples that were of adequate quality were then sequenced. A total of 500 ng of RNA was quantified by Qubit measurement (Thermo Fisher). RNA was then converted to cDNA and ligated to sequencing adaptors with Illumina TriSeq stranded total RNA library preparation (Illumina, San Diego, CA, USA). cDNA samples were then sequenced with the Illumina HiSeq 4000 genome sequencing platform using 150 bp paired-end SBS chemistry. All sequencing was performed at the Genome Facility at the University of Iowa Institute of Human Genetics (IIHG).

STAR was used to align the RNA-seq reads to the human reference genome (version hg38) [30]. We then created BAM files after alignment. FeatureCount was used to measure gene expression [31]. The DESeq2 package was used to import, normalize, and prepare the gene counts for analysis [32]. Gene expression and miRNA expression were independently used for the association analysis. ENSEMBL was used to annotate single exons within the gene expression alignment analysis. Exon expression was then evaluated using the DEXSeq package [33]. BAM files for each sample were used for SNV discovery and base-calling against the human genome reference utilizing SAMtools and BCFtools for sorting and indexing. Results were filtered for duplicates, known non-synonymous single-nucleotide variants, and synonymous variants, and then annotated with ANNOVAR. Gene CNV was estimated using SAMtools and superFreq [34]. BAM files were then used to identify lncRNA, as described previously [35,36]. Lastly, fusion transcripts were determined using the STAR-Fusion suite from fastq files [37]. Supplementary Figure S1 depicts each program used for RNA processing and the identification of various genomic components.

Genomic DNAs (gDNAs) were purified from frozen tumor tissues using the DNeasy Blood and Tissue Kit according to the manufacturer’s (QIAGEN) recommendations. Yield and purity were assessed on a NanoDrop Model 2000 spectrophotometer and by horizontal agarose gel electrophoresis. DNA methylation was determined by using bisulfite-converted gDNA processed on MethylationEPIC arrays. Details of this process have been previously published by our group [38]. The Illumina Infinium MethylationEPIC BeadChip Kit (Illumina) quantifies more than 850,000 methylation sites. Bisulfite-converted samples were denatured and neutralized, then isothermally amplified overnight. The product was fragmented enzymatically. Fragmented DNA was precipitated, resuspended, and placed onto the Illumina methylationEPIC BeadChip and hybridized. The BeadChip was washed, extended, and stained. The arrays were scanned with the Illumina iScan and methylation intensity was measured. Analysis was performed using the Minfi R statistical package [39].

### 2.3. Statistical Analysis

Only baseline clinical characteristics before surgery were included in the statistical analysis. Most genomic data were used as continuous variables, except SNV and fusion genes, which were used as dichotomous variables. To select those variables most informative for the prediction of cytoreduction, we used univariate analysis with ANOVA (*p* < 0.05), as implemented by the caret R package and detailed previously [28]. Significant predictive variables were then used in a multivariate lasso regression prediction model (statistical learning). Thus, poorly annotated variables were removed from model construction.

*Creation of prediction models of cytoreduction with statistical learning:* Significant variables from the univariate analysis were then incorporated into multivariate lasso regression prediction models of optimal and complete cytoreductive surgery. Initial models included only significant variables from one category of clinical or genomic data (i.e., lncRNA expression, miRNA expression, CNV, etc.). Variables were then progressively combined to create more complex prediction models. Multivariate prediction models were fit with the least absolute shrinkage and selection operator (lasso) as implemented in the glmnet R package [40], and detailed previously [28]. Performances of prediction models were measured with the area under the receiver operating characteristics curve (AUC) and 95% CI, and estimated with 1000 replicates of ten-fold cross-validation to avoid over-fitting. Bias-corrected and accelerated bootstrap CIs were computed for each model. The AUC of 0.5 indicates no predictive ability of a model and 1.0 represents perfect predictive performance.

*Validation of predictive models:* HGSC data from TCGA were used to validate the created predictive models [41]. We included only patients within the TCGA database that had at least 6 months of clinical follow-up, adequate data on disease status, and treatment received, including surgical outcome [42]. As with UI patients, only baseline clinical characteristics were included in the validation. Some clinical data were not available in TCGA patients, as this database was not designed for this particular study. Moreover, DNA methylation analysis in TCGA was performed with an earlier chip with fewer features, the Illumina Infinium HumanMethylation27K BeadChip arrays [42]. Some of the significant features from prediction models containing DNA methylation were not able to be validated. BAM files from these HGSC TCGA patients underwent the same analysis pipeline as described previously in the genomic analysis.

Prediction models of cytoreduction constructed with UI data were validated in the TCGA HGSC dataset. When there were missing data in TCGA (either clinical or genomic), we constructed alternative models from our dataset with those variables available in TCGA. The R package, pROC was used to determine thresholds for our model when applied to the TCGA dataset [43]. Models yielding a sensitivity > 90% were ranked from highest to lowest sensitivity, negative predictive value, and AUC. A sensitivity threshold of over 90% will identify most of the patients that will achieve cytoreduction, with improvement over clinical algorithms. The best performing parameters were used to fit a final score of that model to the entire TCGA cohort [28]. Performances measured by AUC between 0.8 and 0.9 were considered ‘very good’; performances between 0.9 and 1 were considered ‘excellent’.

*Validation of predictive models with machine learning methods:* For validation of the best prediction models of cytoreduction in a machine learning platform, we used TensorFlow [44] in a Jupyter notebook with a Keras application programming interface (API) [45]. The TensorFlow code was modified from a tutorial [46]. Training, validating, and testing were performed to account for weights of the outcomes as well as for unbalanced data (mainly for complete vs. optimal patients).

## 3. Results

Of 470 patients within our database, 405 patients were determined to have advanced-stage HGSC. Of these, 273 underwent optimal cytoreduction and 132 received suboptimal cytoreduction. Patients with inadequate clinical data were excluded and we extracted DNA and RNA from the remainder. A total of 83 patients had adequate clinical data, successfully analyzed DNA methylation data, and adequate RNA extraction. A total of 52 of the 83 underwent optimal cytoreductions, 11 of which were complete, and 31 received suboptimal surgeries (Figure 1).

Pre-operative, intra-operative, and postoperative clinical data were compared between the optimal, complete, and suboptimal groups. Significant differences between clinical characteristics for all groups are detailed in Table 1. We computed differences between clinical features for the whole UI cohort of advanced-stage HGSC patients (left side of the table) and those with genomic, RNA-seq, and DNA methylation analyses (middle and right side of the table). There was no difference in the proportion of patients with optimal surgery between the whole cohort and the subgroup that underwent genomic analysis (chi-square *p*-value = 0.402).

**Figure 2:**
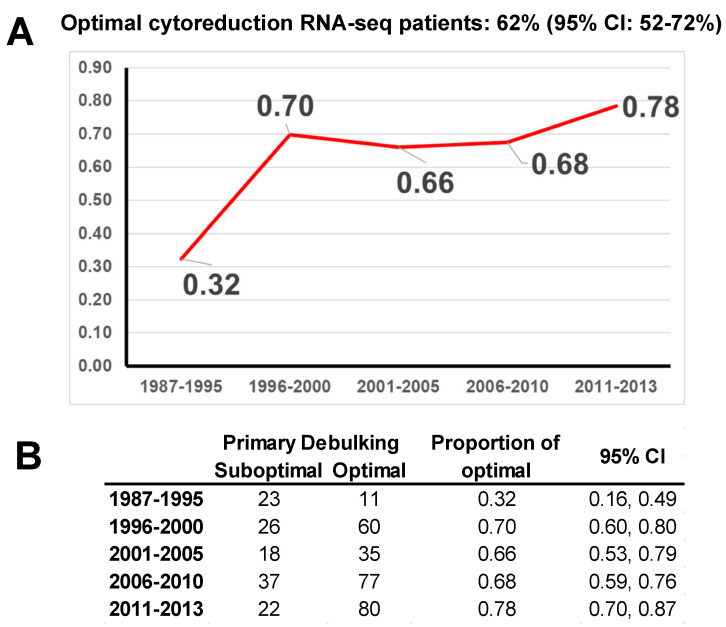
Patients with optimal surgical outcomes at the University of Iowa from 1987 to 2013: (**A**). There has been a steady increase in optimal surgical outcomes during the study period, probably associated with improvements in operative and perioperative care. This improvement was statistically significant (*p*-value < 0.001). (**B**). Proportions and 95% confidence intervals (CI) of optimal cytoreductive surgery by calendar year intervals. In the last available interval, 78% of patients underwent optimal debulking (95% CI: 70%, 77%).

As a reference, we determined the rates of optimal cytoreduction at the UI since the establishment of the Biobank. Starting in 1985, the rate of optimal cytoreduction steadily increased. The 2011–2013 time period demonstrated a rate of optimal cytoreduction of 78%, with a 95% CI of 70% to 87% (Figure 2). Any prediction model that we should build will have to be above this 95% CI (87%) threshold to be considered an improvement. Moreover, based on 95% CIs, there have not been significant changes in optimal surgical resection rates since 1996.

### 3.1. Model Construction

The univariate analysis with ANOVA selected those variables, which were more predictive of optimal or complete surgery. Supplementary Table S1 represents the variable reduction achieved with this initial univariate step. Only clinical data and fusion transcripts did not require this initial step. Significant genomic variables after the initial univariate step are depicted by heatmaps in Supplementary Figure S2. Heatmaps are separated by the surgical outcome.

**Figure 3:**
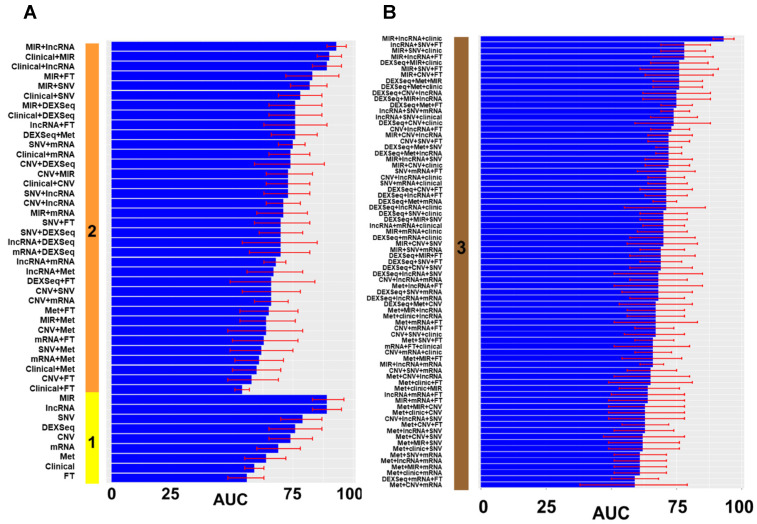
Performance of prediction models of optimal cytoreduction. (**A**). The solid vertical bar represents the number of types of data: **1** (yellow): only one variable was included in the model; **2** (orange): a combination of 2 types of variables. (**B**). Panel with combination of 3 types of variables: solid vertical line with **3** tags (maroon). Different performances on both panels are displayed in ascending order. The x axis is the AUC as a percentage (0–100%). The red error mark displays the 95% confidence interval (CI). Overall, 120 models with different combinations of data were tested. FT: fusion transcripts; Met: DNA methylation; SNV: single nucleotide variation; CNV: gene copy number; DEXSeq: exon expression; lncRNA: long non-coding RNA; MIR: micro RNA, mRNA: gene expression. Graphics were generated with R package *ggplot*.

We created 124 prediction models of optimal cytoreduction using the significant variables from the univariate analysis. The first set of models used only one type of genomic or clinical information. Supplementary Table S2 shows the resulting variables after the multivariate lasso regression analysis in models of prediction, including only one type of datum. Then, we combined different types of data to create models with two and then three categories of data (Figure 3). Models in the figure are ordered by types of data included (y axis) and by the performance of the model, measured in AUC and its 95% CI (x axis). Models with more types of data (four or more) only increased model complexity without any performance improvement.

**Figure 4:**
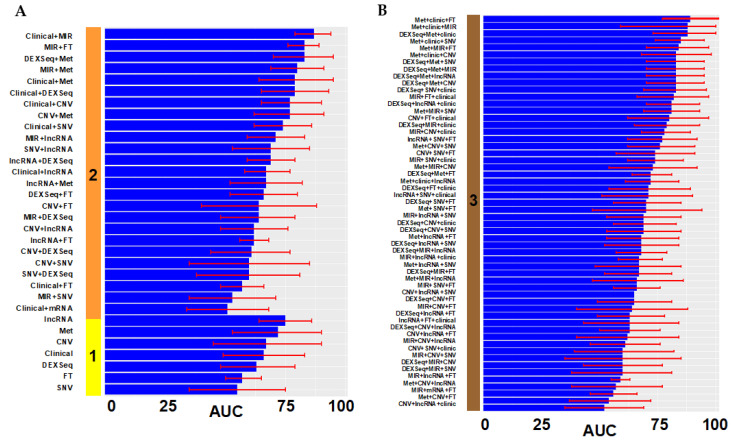
Performance of prediction models of complete cytoreduction. (**A**) The solid vertical bar represents the number of types of data: **1** (yellow): only one variable was included in the model; **2** (orange): a combination of 2 types of variables. (**B**). Panel with combination of 3 types of variables: solid vertical line with **3** tags (maroon). Different performances on both panels are displayed in ascending order. The x axis is the AUC as a percentage (0–100%). The red error mark displays the 95% confidence interval (CI). Overall, 89 additional models with different combinations of data were tested. FT: fusion transcripts; Met: DNA methylation; SNV: single nucleotide variation; CNV: gene copy number; DEXSeq: exon expression; lncRNA: long non-coding RNA; MIR: micro RNA, mRNA: gene expression. Graphics were generated with R package *ggplot*.

We performed the same univariate analysis with ANOVA to select those variables predictive of complete cytoreduction. Then, we introduced significant variables in the univariate analysis (*p* < 0.05) in multivariate lasso regression models to predict complete debulking. It resulted in 89 prediction models of complete cytoreduction using the same methods as for our optimal surgery models (Figure 4).

### 3.2. Model Validation

All models, both for optimal and complete cytoreduction, were then validated using data from the TCGA database. Not all variables for all types of data were available in TCGA. Therefore, we constructed new models from UI data with all variables that were available in TCGA and then we validated all models. The AUC 95% CI of UI initial prediction models for optimal cytoreduction overlapped with validated TCGA models 57% of the time (Supplementary Figure S3). Within the complete cytoreduction models, confidence intervals of the original models and models made for TCGA overlapped 72% of the time after adjusting for the missing data in TCGA (Supplementary Figure S4). Moreover, 66 of the complete debulking models were successfully validated in TCGA. Validated models with similar performances measured by the AUC 95% CI allowed us to further select those models that will be more robust across different datasets.

**Figure 5:**
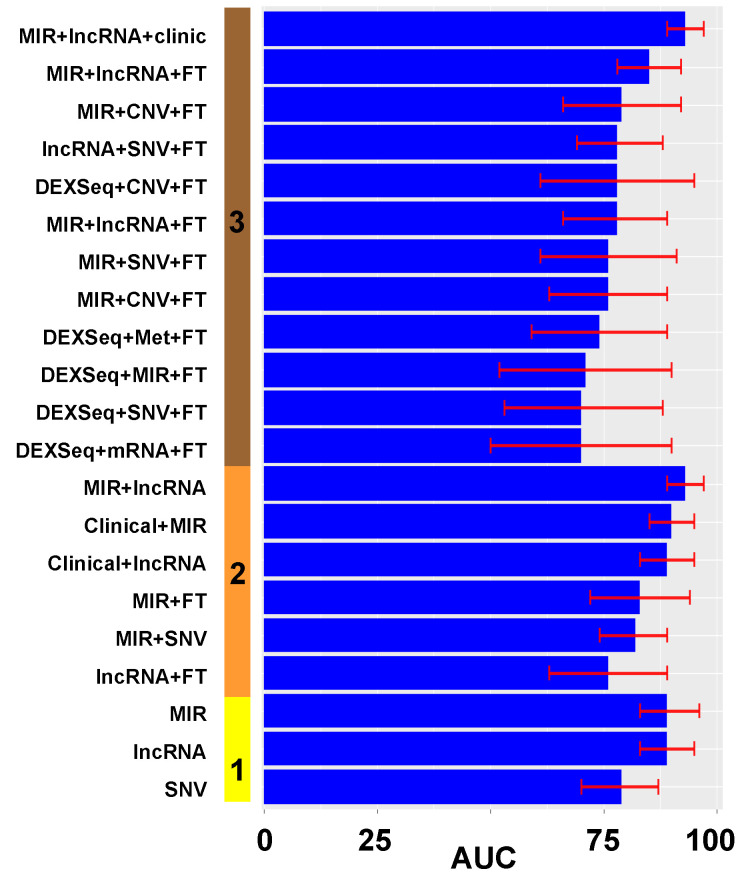
Performance of prediction models with selected variables from the best initial models in optimal cytoreduction. The solid vertical bar represents the number of types of data: **1** (yellow): only one variable was included in the model; **2** (orange): a combination of 2 types of variables; **3** types of variables: solid vertical line with **3** labels (maroon). Different performances are displayed in ascending order. The x axis is AUC as a percentage (0–100%). The red error mark displays the 95% confidence interval (CI). We included all prediction models with a 95% CI of AUC that was included or was superior to 87%. Overall, 21 were selected (that had the potential to be superior to the clinical assessment). FT: fusion transcripts; Met: DNA methylation; SNV: single nucleotide variation; CNV: gene copy number; DEXSeq: exon expression; lncRNA: long non-coding RNA; MIR: micro-RNA, mRNA: gene expression. Graphics were generated with R package *ggplot*.

Overall, in 21 different prediction models of optimal cytoreduction, the 95% CI of the AUC included or was over 87%, which is the upper limit of the 95% CI of our historical rate of cytoreduction. These models are represented in Figure 5, with AUC values and 95% CI on the x axis, and types of data on the y axis. Fusion transcript expression (FT) was the most common category within these models, utilized in 13 of the 21 (62%) models. MicroRNA was the second-most common, present in 12 of 21 (57%) models, followed by lncRNA, 8 out of 21 (38%). The best models included MIR expression, lncRNA expression, and/or clinical data.

**Figure 6:**
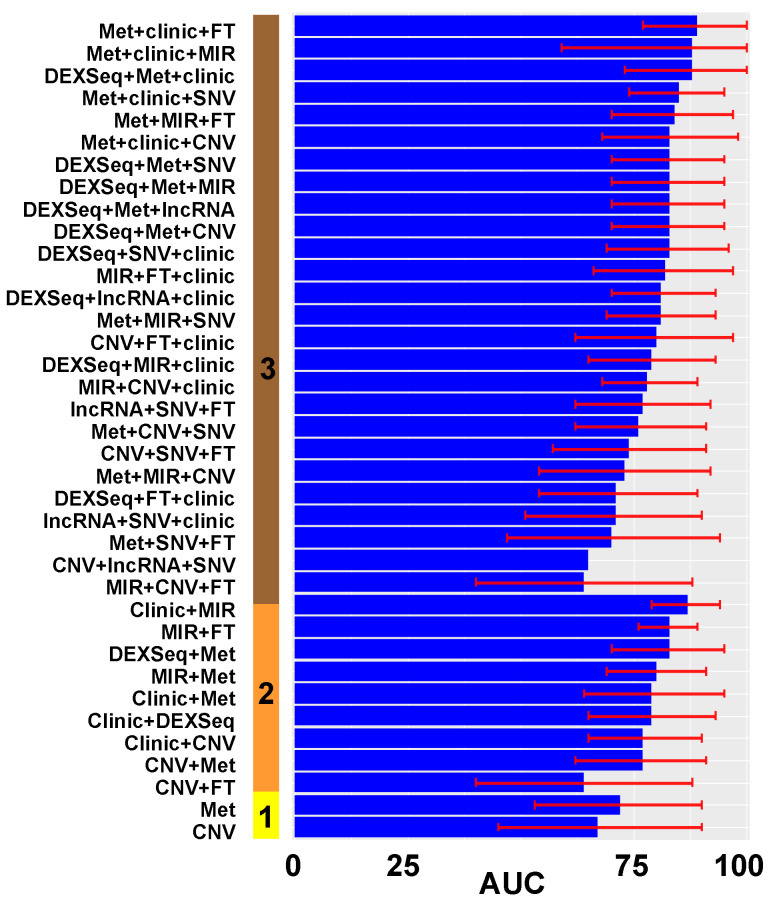
Performance of prediction models for complete cytoreduction with selected variables from best initial models. The solid vertical bar represents the number of types of data: **1** (yellow): only one variable was included in the model; **2** (orange): a combination of 2 types of variables; **3** types of variables: solid vertical line with **3** labels (maroon). Different performances are displayed in ascending order. The x axis is AUC as a percentage (0–100%). The red error mark displays the 95% confidence interval (CI). We included all prediction models with a 95% CI of AUC that was included or was superior to 87%. Overall, 37 were selected (that had the potential to be superior to the clinical assessment). FT: fusion transcripts; Met: DNA methylation; SNV: single nucleotide variation; CNV: gene copy number; DEXSeq: exon expression; lncRNA: long non-coding RNA; MIR: micro-RNA, mRNA: gene expression. Graphics were generated with R package *ggplot*.

In the complete cytoreduction models, 37 had 95% CI’s that crossed the 87% threshold (Figure 6). A total of 19 of the 37 (51%) best-performing models contained DNA methylation (Met) data and 16 of 37 (43%) contained clinical data.

The best-performing models predicting optimal and complete cytoreduction were validated in a machine learning analytical platform using TensorFlow. All machine learning models, performed very well, with the AUC ranging from 89% to 100% (Supplementary Figures S5 and S6).

## 4. Discussion

Optimal cytoreduction is consistently one of the strongest predictors of overall survival in the platinum-based chemotherapy era [9,10,11,12,13,47]. There are several clinical factors known to affect the extent of surgical debulking, including surgical training and patient’s performance status [13,15]. In the present study, we also added biological or genomic factors that predicted cytoreduction accurately. Precise prediction of optimal surgical outcomes with preoperative data has been an elusive objective, leading to suboptimal cytoreduction in 20–30% of surgeries. Additionally, while neoadjuvant chemotherapy has been shown to be non-inferior to primary debulking, there is evidence to indicate that: (a) patients who undergo primary debulking with no residual disease may have improved survival and; (b) those with residual disease ≤ 1 cm may have equivalent survival compared to patients who receive neoadjuvant chemotherapy and have no residual disease at the completion of their surgery [48]. Given this information, it is imperative to identify those patients who can be successfully cytoreduced and triage them to primary cytoreductive surgery, if they are fit for the planned procedure.

Previous prediction models have had minimal to modest success in improving preoperative ability to predict optimal cytoreduction. These models have used such pre-operative criteria as the physical exam, CA-125 and HE-4 levels, CT scan evaluation with artificial intelligence, and ECOG performance status [19,20,21]. These methods generally fail to improve successful optimal cytoreduction beyond 70%. While the laparoscopic scoring system, proposed by Fagotti et al., and refined by Fleming et al., improves optimal cytoreduction rates to 88%, it still requires that the surgeon be prepared to perform an extensive cytoreduction, requiring the allocation of a significant amount of operating room time and resources [22,49]. Additionally, it commits the patient to a surgical procedure, which, while low-risk, is not devoid of it. Ideally, this decision can be made without the cost and resource allocation of a surgical procedure. Moreover, while a recent National Cancer Database (NCDB) study from 2004 to 2015 determined that 73% of patients with advanced stage epithelial ovarian cancer in cancer centers around the US underwent primary cytoreduction surgery [48], only 23% (111 out of 488) of patients undergoing the laparoscopic scoring system would be offered a surgical cytoreductive procedure [22]. It seems that the improved rate of surgical outcomes with this laparoscopic algorithm may be at the expense of a large proportion of patients not being offered primary cytoreduction.

Artificial intelligence (AI) has the promise of enhancing prediction modeling. AI has already been applied in assessing CT imaging as prognosticator and predictor of outcomes for ovarian cancer [50,51,52]. However, the question remains if CT imaging processed throughout AI improves the capability of predicting which patients will undergo optimal surgery. Advanced ovarian cancer is very heterogenous and addressing all metastatic diseases in the abdomen is challenging for any processing system (human or machine). So, areas underestimated by any system may have clinical negative consequences. Once AI modeling can identify automatically (by segmentation algorithms) cancerous tumors, modeling methods may achieve high accuracy. Additionally, machine learning analytics may also improve the integration and prediction of diverse data (analyzed or not with AI), such as clinical, radiomics, genomics, and environmental data, thus reaching performance levels that are worth using clinically (an AUC over 95%). Until then, clinical decision-making remains the standard.

Previous attempts to use genomic data to create prediction models for cytoreduction have had mixed success [24,25]. In our study, we created 21 models to predict optimal cytoreduction and 37 to predict complete cytoreduction integrating genomic and clinical data. The 95% CIs of all these models met the threshold established to improve on clinical criteria for surgical outcomes. This means that, following future prospective validation, these models have the potential to predict optimal cytoreduction with higher accuracy than the clinical decision-making of an experienced gynecologic oncology team. Additionally, prediction models of complete cytoreduction also had excellent performance, measured by the AUC, and were comparable to those of optimal debulking. These models were also validated within the TCGA database, meaning that they are capable of performing well in independent datasets. Moreover, these models were validated with a different analytical platform (machine learning), suggesting that they are also precise in measuring the outcome.

One criticism of primary cytoreduction is the increase in morbidity and mortality inherent in an aggressive surgical procedure. Narasimhulu et al. developed a prediction tool that significantly reduces perioperative morbidity and mortality by using simple selection criteria to determine which patients would best tolerate upfront debulking [53]. By reducing this morbidity, it improves the rate of optimal outcomes in primary surgery. The clinical criteria used for this tool include age, performance status, albumin, surgical complexity, and stage. We incorporated these significant predictors of morbidity into the clinical portion of our prediction model with the exception of albumin, which was not reliably available in our cohort.

Each model incorporated different combinations of clinical and genomic data. Within the optimal cytoreduction models, three included only one category of data. Fusion transcripts and miRNA expression were the most common categories represented, present in 62% (13/21) and 57% (12/21) of the models, respectively. The two best-performing models with the smallest confidence intervals incorporated miRNA and lncRNA with and without clinical data. Models solely using miRNA and lncRNA were also high-performing. miRNA and lncRNA are known to be significant regulators in ovarian cancer, correlated with overall survival, and are relatively new categories of investigation for novel therapeutics [34,51,52]. We are only beginning to understand the extent of their importance.

Within the complete cytoreduction models, DNA methylation data were the most frequently represented category of data in the 37 models, which have the potential to confirm our hypothesis. Our group has previously shown that DNA methylation status is correlated with optimal and suboptimal cytoreductive outcomes in ovarian cancer [38]. While that is certainly intriguing, our models were created based on just 11 patients with R0 outcomes and, therefore, it is likely that more specimens will be needed to refine these models. However, complete and optimal cytoreduction models taken as a whole, confirm our hypothesis that tumor biology can be used to inform our surgical decision-making.

The strengths of our study include a comprehensive analysis of genetic and epigenetic data on a single institution cohort of HGSC patients, treated with a similar philosophy and with clinical and outcomes data available. The involvement of a vast array of genomic data provides a complete representation of the genome and its regulators to the extent of our current knowledge. We created models for both complete and optimal surgical outcomes. Moreover, the resulting models were validated in an independent, comprehensive clinical-genomic database, TCGA; it informed which models were more robust and consistent in order to further validate prospectively.

Moreover, we recently published an assessment of overall survival in the patients found in the UI Biobank demonstrating that patients who undergo optimal cytoreduction have improved overall survival, regardless of their subsequent response to chemotherapy [54]. This solidifies the theory that improving optimal or complete cytoreduction rates in our patients will subsequently lead to improvements in overall survival. While that is established in the literature at large, proving the point in the database used in this study further strengthens our conclusions.

There are several limitations to our study. This was a retrospective study and, thus, was subject to some biases, particularly selection bias. Patients were chosen for surgery based on traditional clinical decision-making and this subset of patients were the ones included in our models. This could have affected our models since these were the patients in whom surgery was already expected to be successful, creating a cohort of patients with a higher probability of an optimal outcome. In order to correct for this bias, prospective validation of our models must be performed using all patients assessed for cytoreduction. Another limitation is that our study was conducted with patients from a single institution. Ovarian cancer populations can have significant genomic variation and, therefore, our models may only be valid within the University of Iowa population [55]. Therefore, other institutions may require further refinement for these models to fit their patient populations. We chose the definition of optimal cytoreduction (≤1 cm) in agreement with GOG usage in multiple trials [3,4,5,6,7,8]. There is a linear association between the size of the maximum residual disease and overall survival [11], but to create a classifier or predictor, a dichotomous outcome for the model is preferred [56]. Although all surgeons aspire to achieve complete cytoreduction, a classifier that predicts optimal cytoreduction to ≤1 cm has been used as inclusion/exclusion criteria for clinical trials extensively in the past, as previously noted.

Lastly, there was also the concern that the resulting models would not translate into a simple test that could be performed easily, promptly, and inexpensively in clinical settings before surgery. The best resulting models contained 2–3 types of genomic or clinical information. All these genomic parameters could be assessed by PCR-based analytical tests. PCR-based tests are cheap, fast, and can be performed from small biological samples. Our study was performed in samples from a historical biobank, with flash frozen tissue that may have degraded over time. We expect to have better yields from fresh samples extracted exclusively to be applied to the model. In other gynecological tumor models, there was a high degree of concordance of molecular parameters and feasibility between pre-operative specimens and surgical specimens [57]. We expect to see the same concordance with pre- and post-surgical HGSC specimens.

## 5. Conclusions

In summary, the results of our study are encouraging, despite some limitations due to the study design that biological data can be used to predict surgical outcomes in advanced high-grade serous ovarian cancer. Further work is needed to prospectively validate these models and to ascertain whether selection biases affected prediction model performances. Prospective validation in consecutive HGSC patients, using fresh tumor samples, will further validate and select the best-performing model, while optimizing it for rapid and inexpensive processing. Finally, this diagnostic tool could be assessed in a diagnostic trial, where specimens are collected before surgery to inform clinicians about potential cytoreduction options based on the performance. We foresee that this diagnostic tool will help surgeons to improve surgical outcomes, by increasing complete and optimal cytoreduction rates and decreasing surgical morbidity, while still including the majority of advanced-stage HGSC patients.

## Figures and Tables

**Table 1:** Patient Characteristics and Association with PCS Outcome.

		All UI HGSC Population			Samples with RNA-seq and DNA Methylation		
		Optimal (*n* = 273)	Suboptimal (*n* = 132)	*p*-Value	Optimal (*n* = 52)	Suboptimal (*n* = 31)	*p*-Value
Preoperative characteristics	Age (mean)	61	61	0.742	62	58	0.204
	BMI (mean)	28.3	28.1	0.772	25.5	27.8	0.149
	Charlson Morbidity Index **						0.604
	Low (1–3)	44	19	Ref	8	6	Ref
	Medium (4–6)	182	87	0.738	31	16	0.548
	High (>6)	37	19	0.660	5	3	0.806
	Preop CA-125 (mean)	1613	2067	0.224	2695	3030	0.808
	Disease in Upper abdomen (Other than Omentum) by Imaging	203	101	0.638	34	20	0.936
	Large bowel	14	8	0.698	1	1	0.711
	Spleen	5	0	0.982	0	0	-
	Mesenteric LN	15	7	0.936	2	1	0.884
	Porta/Hepatis	21	17	0.097	2	1	0.884
	Ascites (upper abdomen)	115	56	0.954	20	8	0.241
	Other	90	52	0.204	12	11	0.225
	Disease in the Chest by Imaging	25	29	<0.001 *	1	4	0.077
	Tumor	7	19	0.026 *	1	2	0.313
	Pleural effusion	25	27	0.002*	1	2	0.313
	Neoadjuvant chemotherapy	22	9	0.700	10	4	0.450
Operative characteristics	FIGO Stage			0.001 *			0.225
	III	224	89		40	20	
	IV	49	43		12	11	
	Hysterectomy	210 (77%)	79 (60%)	<0.001 *	38 (73%)	18 (58%)	0.161
	Surgery to remove cervix	188 (69%)	63 (48%)	<0.001 *	32 (62%)	13 (42%)	0.085
	Adnexectomy	263 (96%)	125 (95%)	0.443	51 (98%)	29 (94%)	0.313
	Omentectomy	260 (95%)	118 (89%)	0.031 *	49 (94%)	29 (94%)	0.894
	Surgery large bowel	96 (35%)	35 (27%)	0.082	16 (31%)	9 (29%)	0.868
	Surgery small bowel	5 (2%)	3 (2%)	0.765	0 (0%)	1 (3%)	0.991
	Splenectomy	4 (1%)	0 (0%)	0.984	1 (2%)	0 (0%)	0.992
	Diaphragmatic stripping	8 (3%)	0 (0%)	0.977	5 (10%)	0 (0%)	0.992
	Para-aortic lymphadenectomy	56 (10%)	13 (21%)	0.009 *	3 (6%)	3 (10%)	0.510
	Residual disease			0.981			0.988
	Microscopic	71	0		11	0	
	Macroscopic	202	132		41	31	
	Surgical complexity index ^#^						
	Low	126	91	Ref	30	20	Ref
	Intermediate	140	41	<0.001 *	20	11	0.685
	High	7	0	0.978	2	0	0.992
Outcomes	30-day mortality	4 (1.5%)	3 (2.3%)	0.558	0 (0%)	0 (0%)	N/A
	90-day mortality	8 (2.9%)	9 (6.8%)	0.071	0 (0%)	1 (1%)	0.991

* Statistically significant. ** Charlson Comorbidity Index is a measure of the prognostic burden of all associated morbidities to predict mortality and is the most validated measure of the prognostic impact of multiple chronic illnesses [26]. ^#^ Surgical complexity score: score to predict surgical morbidity and 90-day mortality after primary debulking surgery for HGSC [27].

## Data Availability

Data for the prediction model were submitted to the GEO at NCBI website [58]. Datasets with methylation data can be browsed by their accession number: GSE133556. Datasets with RNA-seq can be browsed by their accession number: GSE156699. The validation portion of this study was performed in silico, with de-identified publicly available data. All data from TCGA are available at their website [59]. Software utilized by this study is also publicly available at the Bioconductor website [60].

## Supplementary Materials

The following supporting information can be downloaded at: https://www.mdpi.com/article/10.3390/cancers14143554/s1. Supplementary Figure S1: Pipeline of genomic analytics starting with RNA sequencing; Supplementary Table S1: Variable selection and variables after prediction model construction with type of data; Supplementary Figure S2: Heatmap of selected variables after univariate ANOVA analysis; Supplementary Table S2: Resulting significant variables after multivariate lasso regression analysis and construction of predictive models–Optimal cytoreduction; Supplementary Figure S3: TCGA validation of prediction models of response in optimal cytoreduction; Supplementary Figure S4: TCGA validation of prediction models of response in complete cytoreduction; Supplementary Figure S5: Validation of optimal cytoreduction prediction models with machine learning analytical platform; Supplementary Figure S6: Validation of complete cytoreduction prediction models with machine learning analytical platform.
