# Supplementary material for: Integrated Clinical and Genomic Models to Predict Optimal Cytoreduction in High-Grade Serous Ovarian Cancer
Source: Cancers (Basel). 2022 Jul 21;14(14):3554. doi: 10.3390/cancers14143554 (PMC9323510; doi:10.3390/cancers14143554)
Supplement: Supplementary file 1 [file cancers-14-03554-s001.zip › cancers-1774657-supplementary.pdf]

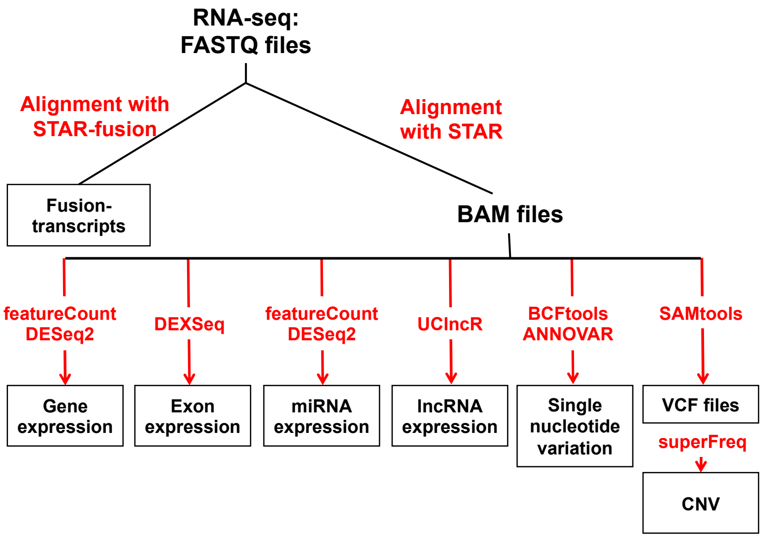

Supplementary Figure S1: Pipeline of genomic analytics starting with RNA sequencing. From fastq files originated from RNA-seq, we created fusion transcripts and BAM files. The rest of genomic elements were produced from these BAM files and different software analytics. In red, different software utilities used to generate genomic elements for this project.

Supplementary Table S1: Variable selection and variables after prediction model construction with type of data.

Type of Data	Initial Number of variables	Variables after selection: univariable ANOVA analysis with k-fold cross-validation*	Variables also present in TCGA dataset
Gene expression: mRNA	23,528	496	496
miRNA expression	1,914	16	16
Gene copy number	23,443	3,477	3,477
Individual exon expression	468,562	9,290	7,402
Single nucleotide variation	13,840	859	770
DNA methylation	66,042	4,932	2,433
Long non-coding RNA	16,325	473	417
Fusion transcripts	597	142*	6
Clinical	40	1*	0

To reduce the number of variables, we used univariate analysis of all data with ANOVA to select the variables that were more informative for prediction of response, with a $p\text{-value}<0.05$ (3rd column). All these informative variables found in the UI dataset were available in TCGA for validation in the first 4 types of data, but not for the rest. In the last column we describe the number of variables also present in TCGA. Validation analyses were performed with these common variables to both datasets.

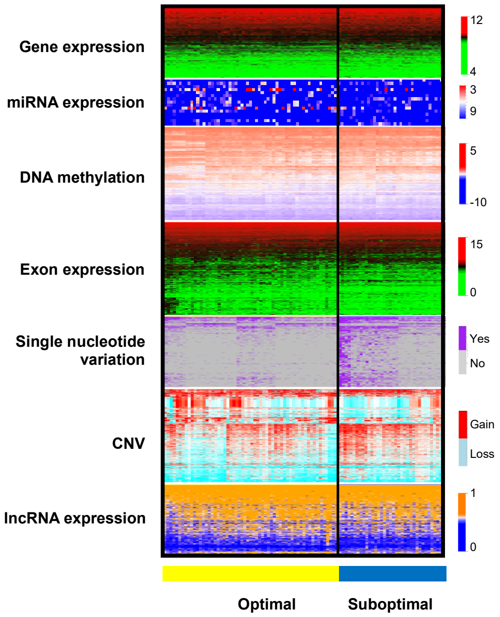

Supplementary Figure S2: Heatmap of selected variables after univariate ANOVA analysis. Representation of the significant variables after univariate analysis (p<0.05) for different types of genomic data: gene, miRNA, exon, and long non-coding RNA (lncRNA) expression, DNA methylation, single nucleotide variation, and gene copy number variation (CNV). At the right side of each heatmap there are color-coded range of values for all genomic variables. Fusion-transcripts presence did not require dimension reduction (initial # of variables: 597), and neither did clinical data (initial # of variables: 40) .

Supplementary Table S2: Resulting significant variables after multivariate lasso regression analysis and construction of predictive models – Optimal cytoreduction

		Step 1	Step 2	
**Type of Data**	**Initial # variables**	**Univariable ANOVA**	**Multivariable lasso**	**Final variables**
**Clinical**	40	-	1	Disease in chest by imaging
**Gene expression**	23,528	496	9	*GBP1P1, ACTA1, N4BP2L1, ZNF426, MACROD2-IT1, BIRC7, UBA7, SLC26A2, MTFR1*
**miRNA expression**	1,914	16	16	MIR5008, MIR603, MIR3685, MIR1251, MIR3657, MIR301A, MIR644A, MIR99A, MIRLET7C, MIR650, MIR1255A, MIR4804, MIR4639, MIRLET7A1, MIR2278, MIR3978
**Gene copy number variation**	23,443	3,477	49	*NMNAT1, FBXO44, KIF17, CDC20, HYI, DEPDC1, MRPS14, RNF2, ARF1, NDUFS1, TBC1D5, VILL, TBC1D23, NFKBIZ, CCDC54, NMU, COL4A3BP, SEMA6A, PGBD1, AKR1D1, DPP6, LOXL2, C9orf64, NR6A1, NUP188, FRMPD2, SLC29A2, TPCN2, BIRC2, FKBP4, DDX47, SSPN, GLT1D1, NEK3, SUPT16H, TGFB3, PML, ADCY7, MT2A, CACNA1G, TUBD1, FTSJ3, ANAPC11, ZNF563, SFRS14, ERG, TMEM27, ZNF41, GLUD2*
**Single nucleotide variation**	13,840	859	57	*AADAC, ABHD8, ADAMTS18, ADD1, AKAP8, AKIP1, ANPEP, ARHGAP6, ARSJ, C11orf16, C2orf69, CAPN1, CCDC187, CCDC6, CCNI, CEP128, CNTN4, COCH, COG5, DDX59, DENND1C, DKK3, DNASE1, DOK1, DUSP16, EID2, FBXO16, HYLS1, KCNJ5, KIAA1217, LNPEP, MECOM, NR5A1, NSUN5, NSUN7, PHACTR4, PIK3CA, PKHD1L1, RAB27A, SERPINA3, SH2D3C, SHROOM1, SIK2, SMS, TAF9, TAS2R20, TDP2, TMEM144, TMEM160, TMEM182, TRIM38, TUBD1, UCKL1, ZBBX, ZDHHC14, ZNF283, ZNF429*
**DNA methylation**	66,042	4,932	26	*GLTPD1, CDC20, HYI, DEPDC1, AADACL2, SUCNR1, CHRNA9, RPS14, LOC100287718, NR6A1, OTUD1, BIRC2, CNOT2, LOC100128233, TGFB3, ARPP19, MIR548H4, ARRDC4, DUSP3, ZNF563, MRPS12, C19orf68, MPST, TMEM27, ZNF41*
**Long non-coding RNA**	16,325	473	77	LINC01778, AC117944.1, AC243547.2, LINC00624, AL160286.2, AC093422.2, FLVCR1-DT, AC074011.1, AC118345.1, LINC01796, NCKAP5-AS2, AC009480.1, LINC01806, AC019197.1, AC012087.2, AC063952.4, AC107027.3, TERC, AC092953.2, AC046143.2, AC226119.1, AC109347.1, AC109927.2, LINC02430, AC020703.1, AC010343.3, AC099520.1, AL365205.3, AL034374.1, AL355297.3, AL109924.5, AL109924.2, AC005014.3, AC019117.2, AC018643.1, AC090186.1, AC100860.1, AF186192.3, AL161729.2, DNAJC9-AS1, AL512656.1,

				AP000753.2, FAM138D, TESC-AS1, AL356752.1, AL356020.1, AC005520.3, AL160313.1, AC100839.1, AC100839.2, AC103740.1, AC105133.1, AC087761.1, AC023302.1, AC021739.3, AC078905.1, AC009097.3, AC022165.1, AC092143.2, AC091153.3, LINC02087, RNFT1-DT, AC110285.3, AC124283.1, LINC02564, AC009802.1, AC008764.7, AC002128.1, AC020922.2, AC012313.3, AL035458.2, AL356652.1, AL035420.3, HAR1A, CU634019.6, LINC00102, ARSD-AS1
**Fusion genes**	597	142	2	MTCH2--AGBL2, NF1--RAB11FIP4
**Exon expression**	468,562	9,290	68	ENSG00000010361:008, ENSG00000067829:032, ENSG00000088727:051, ENSG00000089234:002, ENSG00000091947:002, ENSG00000105483:012, ENSG00000111361:001, ENSG00000112659:064, ENSG00000114446:005, ENSG00000114757:003, ENSG00000121766:019, ENSG00000133318:028, ENSG00000133742:015, ENSG00000133742:020, ENSG00000134256:006, ENSG00000135480:018, ENSG00000140395:006, ENSG00000142208:006, ENSG00000144445+ENSG00000263530:022, ENSG00000148248:017, ENSG00000148843:044, ENSG00000151532:002, ENSG00000153113:104, ENSG00000154328:011, ENSG00000158220:015, ENSG00000166260:018, ENSG00000172638:015, ENSG00000172661:005, ENSG00000173406+ENSG00000162600:066, ENSG00000174004+ENSG00000163964:030, ENSG00000177426:024, ENSG00000184428:031, ENSG00000201109:001, ENSG00000205981:006, ENSG00000220412:001, ENSG00000225214:002, ENSG00000230231:002, ENSG00000231924:007, ENSG00000235098:008, ENSG00000236782:009, ENSG00000237441:030, ENSG00000248210:013, ENSG00000252713:001, ENSG00000256894:005, ENSG00000265907:002, ENSG00000267383+ENSG00000267220:003, ENSG00000269427:001, ENSG00000273472:001, ENSG00000005483:052, ENSG00000105576:064, ENSG00000108950:025, ENSG00000115414:065, ENSG00000122432:001, ENSG00000122786:027, ENSG00000128683:027, ENSG00000133710:044, ENSG00000135406:004, ENSG00000136783:001, ENSG00000163359:036, ENSG00000164070:013, ENSG00000166260:019, ENSG00000168264:002, ENSG00000174718:005, ENSG00000177380:039, ENSG00000177666:013, ENSG00000189143:003, ENSG00000237520:002, ENSG00000257496:002,

*Lasso regression was performed directly with no pre-reduction with ANOVA because the smaller number of variables in two types of data: fusion transcripts and clinical data.

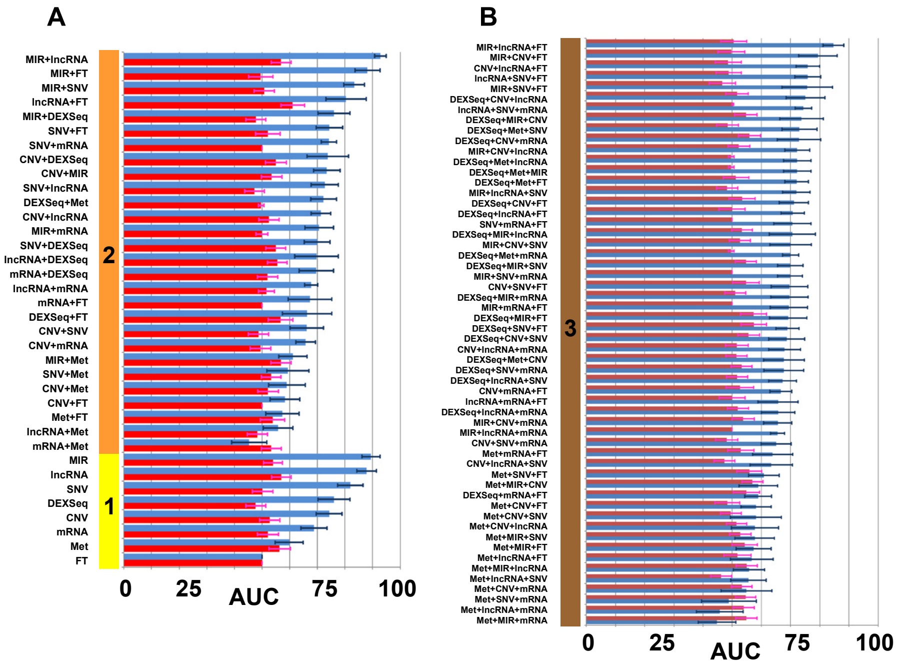

Supplementary Figure S3: TCGA validation of prediction models of response in optimal cytoreduction.

A. The solid vertical bar represents the number of types of data (as per models constructed with UI data): **1** (yellow): only one variable was included in the model; **2** (orange): combination of 2 types of variables. **B.** Panel with combination of 3 types of variables: solid vertical brown line labeled “3”.

Different performances on both panels are displayed in ascending order. The x axis is AUC as a percentage (0-100%). The red error mark displays the 95% confidence interval (CI). Over 57% of TCGA models had an AUC 95% CI that overlapped with AUC 95% CI of UI models. Overall, 93 models were validated in TCGA

FT: Fusion transcripts; Met: DNA methylation; SNV: single nucleotide variation; CNV: gene copy number; DEXSeq: exon expression; lncRNA: long non-coding RNA; MIR: micro RNA, mRNA: gene expression. Graphics were generated with R package *ggplot*.[69]

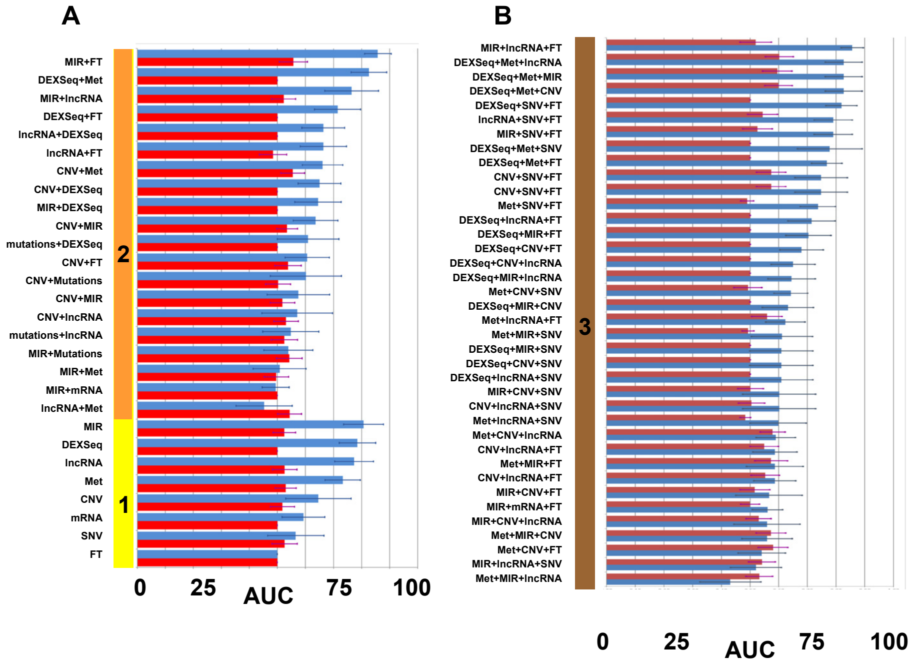

Supplementary Figure S4: TCGA validation of prediction models of response in complete cytoreduction.

A. The solid vertical bar represents the number of types of data (as per models constructed with UI data): **1** (yellow): only one variable was included in the model; **2** (orange): combination of 2 types of variables. **B.** Panel with combination of 3 types of variables: solid vertical brown line labeled "3".

Different performances on both panels are displayed in ascending order. The x axis is AUC as a percentage (0-100%). The red error mark displays the 95% confidence interval (CI). Over 72% of TCGA models had an AUC 95% CI that overlapped with AUC 95% CI of UI models. Overall, 66 models were validated in TCGA

FT: Fusion transcripts; Met: DNA methylation; SNV: single nucleotide variation; CNV: gene copy number; DEXSeq: exon expression; lncRNA: long non-coding RNA; MIR: micro RNA, mRNA: gene expression. Graphics were generated with R package *ggplot*.[69]

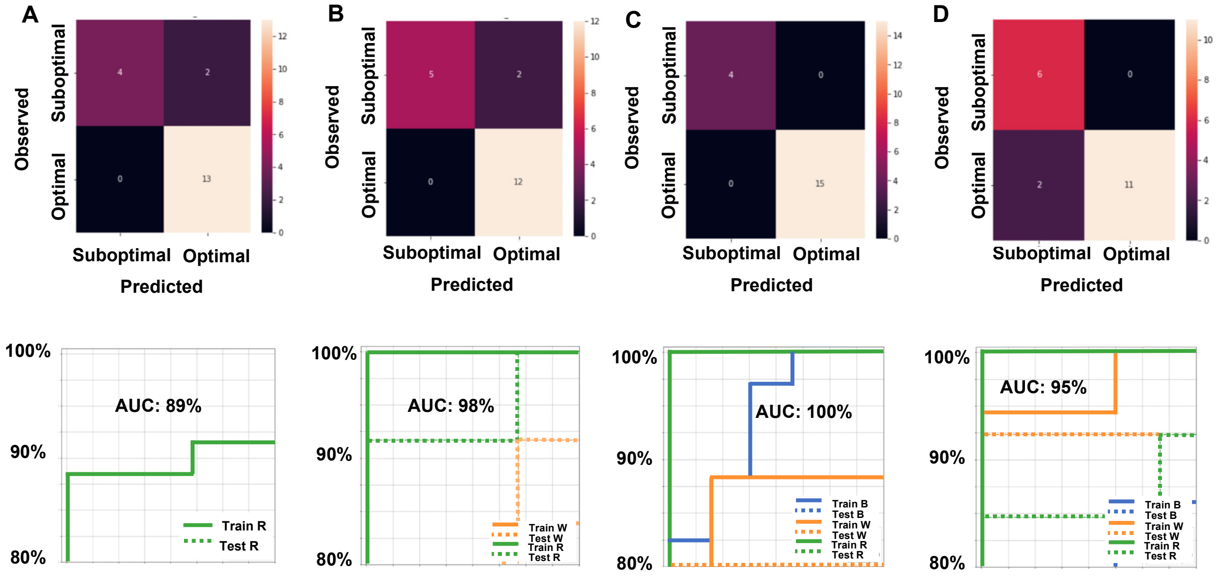

Supplementary Figure S5: **Validation of optimal cytoreduction prediction models with machine learning analytical platform**.

A. Model with micro-RNA (MIR) data. The superior panel shows the confusion matrix representing the observed versus the predicted values. The inferior panel is an ROC graphic: true positives in the x axis, false positives in the y axis, and AUC results. Train R: results of unbalanced (or re-sampling) model training; Test R: results of re-sampling model testing. **B.** Model with clinical and lncRNA data. Superior panel is as before. Inferior panel represents the ROC graphic including: 1) models accounting for weights of the outcome: Train W: results of weighted model training; Test W: results of weighted model testing; 2) models accounting for unbalanced samples: Train R: results of unbalanced (or re-sampling) model training; Test R: results of re-sampling model testing. **C.** Model with MIR and lncRNA data. Superior panel is as before. Inferior panel represents the ROC graphic including: 1) basic model: Train B: results of basic model training; Test B: results of basic model testing; 2) models accounting for weights of the outcome: Train W: results of weighted model training; Test W: results of weighted model testing; 3) models accounting for unbalanced samples: Train R: results of unbalanced (or re-sampling) model training; Test R: results of re-sampling model testing. **D.** Model with clinical, MIR and lncRNA data. Superior panel is as before. Inferior panel represents the ROC graphic including: 1) basic model: Train B: results of basic model training; Test B: results of basic model testing; 2) models accounting for weights of the outcome: Train W: results of weighted model training; Test W: results of weighted model testing; 3) models accounting for unbalanced samples: Train R: results of unbalanced (or re-sampling) model training; Test R: results of re-sampling model testing.

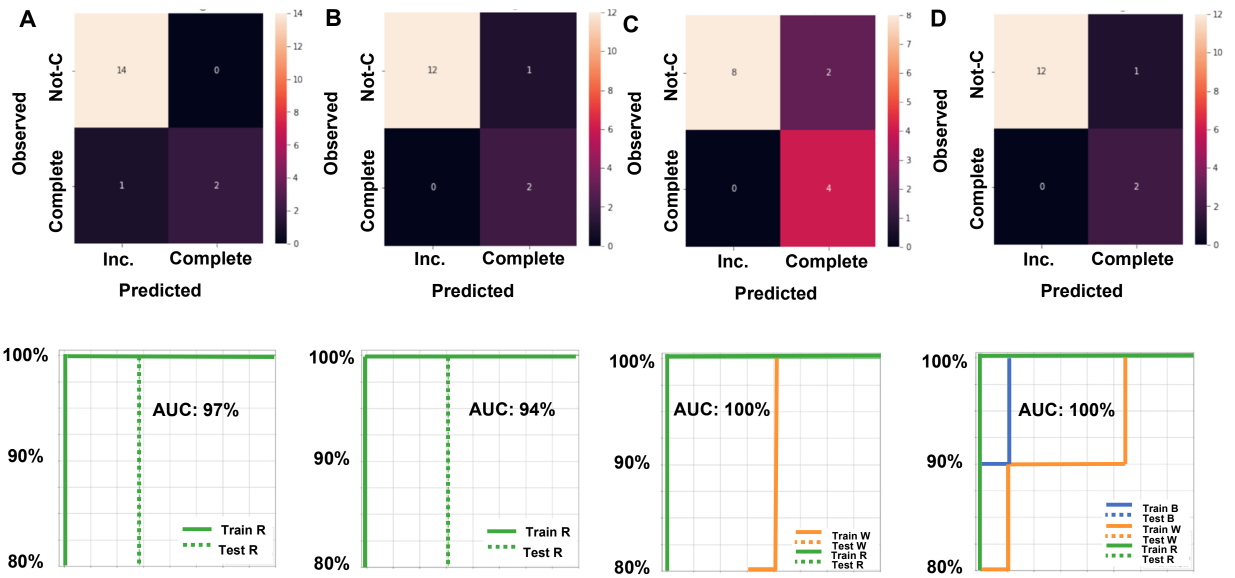

Supplementary Figure S6: **Validation of complete cytoreduction prediction models with machine learning analytical platform**.

A. Model with clinical and micro-RNA (MIR) data. The superior panel shows the confusion matrix representing the observed versus the predicted values. Not-C: Not complete cytoreduction. The inferior panel is an ROC graphic: true positives in the x axis, false positives in the y axis, and AUC results. Train R: results of unbalanced (or re-sampling) model training; Test R: results of re-sampling model testing. **B.** Model with clinical, DNA methylation (MET), and single exon expression (DEXSeq) data. Superior and inferior panels are as before. **C.** Model with clinical, MET, and fusion transcripts (FT) expression data. Superior panel is as before. Inferior panel represents the ROC graphic including: 1) models accounting for weights of the outcome: Train W: results of weighted model training; Test W: results of weighted model testing; 2) models accounting for unbalanced samples: Train R: results of unbalanced (or re-sampling) model training; Test R: results of re-sampling model testing. **D.** Model with clinical, MET, and MIR data. Superior panel is as before. Inferior panel represents the ROC graphic including: 1) basic model: Train B: results of basic model training; Test B: results of basic model testing; 2) models accounting for weights of the outcome: Train W: results of weighted model training; Test W: results of weighted model testing; 3) models accounting for unbalanced samples: Train R: results of unbalanced (or re-sampling) model training; Test R: results of re-sampling model testing.
